# The value of interferon-gamma blood tests for the diagnosis of tuberculosis in HIV patients

**DOI:** 10.1186/1471-2334-13-S1-P1

**Published:** 2013-12-16

**Authors:** Olga Adriana Căliman-Sturdza, Corina Stănescu

**Affiliations:** 1 Emergency County Hospital “New St. John”, Suceava, Romania; 2 “Gr.T.Popa” University of Medicine and Pharmacy, Iaşi, Romania

## Background

Tuberculosis is the most common opportunistic infection in HIV patients in Romania, but the diagnosis is often difficult because of its many atypical forms.

## Objectives

The aim of this study was to evaluate the value of whole interferon-gamma assay, QuantiFERON TB.Gold in Tube (QFT) for the diagnosis of tuberculosis (TB) in HIV patients.

## Methods

We performed QFT in 80 HIV patients with suspected latent or active TB, between January 2008 to December 2010.

## Results

We enrolled in the study 80 HIV patients (10 children and 70 adults); M:F = 36:44. All subjects were previously BCG vaccinated, 24 (30%) had positive family contact and 16 (20%) had a history of tuberculosis. 45 (56.2%) patients were in stage C3 (CD4<200 cells/µL), 32 (40%) in stage C2 (CD4 = 201-400) and 3 (3.8%) patients in stage B1 (CD4>400). We diagnosed 52 (65%) patients with active or latent TB, out of which 2 cases of pleural effusion, 7 miliary, 22 with pulmonary forms, 8 TB meningitis, 2 lymph nodes TB and one intestinal tuberculosis. Tuberculin skin test (TST) was performed in all patients. Sixteen patients were TST positive: only 2 patients in the group with CD4<200, 10 patients in the group with CD4 between 200-400 and 4 subjects with CD4>400. The QFT test was positive in 27 (33.75%) patients, negative in 44 (55%) and indeterminate in 9 (11.25%). We obtained 14 (31.1%) QFT positive results in patients with CD4<200, 10 (31.25%) QFT positive results in group with CD4 = 201-400 and 3 (100%) positive results at patients with CD4>400. In stage C3 (CD4<200) the positive TST tests was significantly lower (4.4%) compared to positive QFT (31.1%).

## Conclusions

The QuantiFERON TB.Gold test is a useful tool for the diagnosis of tuberculosis in HIV patients, even in those in terminal stage; it is more specific than TST, and it could replace TST in the near future.

